# Clinical Trial Evidence on Suicide Risk Assessment and Prevention Strategies: An Analysis of Completed Studies Registered on ClinicalTrials.gov

**DOI:** 10.3390/healthcare14213713

**Published:** 2026-11-02

**Authors:** Ahmed M. Ashour

**Affiliations:** Department of Pharmacology and Toxicology, College of Pharmacy, Umm Al-Qura University, Makkah 715-21955, Saudi Arabia; amashour@uqu.edu.sa; Tel: +966-566664464

**Keywords:** suicide, attempted, suicidal ideation, suicide prevention, suicide risk assessment

## Abstract

**Background/Objectives:** Suicide remains a leading cause of preventable mortality across the globe, fundamentally representing a complex public health challenge requiring effective risk assessment and prevention strategies. Despite increasing research activity, evidence from clinical trials remains heterogeneous, notably with the effectiveness of available interventions substantially varying across populations and settings. Accordingly, a systematic evaluation of completed clinical studies is essential to clarify current research trends, intervention effectiveness, and existing evidence gaps in suicide prevention. **Methods:** In this descriptive analytical study, I reviewed completed interventional clinical trials from ClinicalTrials.gov that investigated suicide risk assessment tools, prevention strategies, and pharmacological and non-pharmacological interventions targeting suicidal ideation or behavior in adult populations, particularly individuals aged 18 years and above. Trials were identified using predefined eligibility criteria. Data were extracted on study design, populations at risk, intervention type, suicide-related outcome measures, and safety reporting. The initial search was done on 28 March 2026 for completed interventional studies using predefined search terms, and the findings were synthesized descriptively without quantitative pooling. **Results:** Eight completed interventional trials met inclusion criteria. Most studies were randomized controlled trials enrolling high-risk adult populations, including individuals with mood disorders, post-traumatic stress disorder, recent psychiatric hospitalization, or prior suicide attempts. Non-pharmacological interventions, particularly cognitive behavioral therapy, collaborative care models, safety planning, and digital or telehealth-based approaches, predominated. Validated suicide risk instruments such as the Columbia-Suicide Severity Rating Scale, Beck Scale for Suicide Ideation, and PHQ-9 item 9 were commonly used, though outcome designation and reporting completeness varied. Registry-reported findings suggested heterogeneous effectiveness across interventions, and safety reporting was inconsistent, with limited long-term data available. **Conclusion:** This registry-based analysis highlights important progress in suicide prevention research while revealing persistent methodological and reporting limitations. The greater standardization of outcome measures, improved result reporting, broader population inclusion, and longer follow-up are needed to strengthen the evidence base and inform effective clinical and policy decision-making in suicide prevention. However, and while registry-based findings show meaningful research activity, incomplete result-posting across several trials limits definitive conclusions regarding comparative efficacy, underscoring the need for improved reporting standardization.

## 1. Introduction

Suicide, a recent World Health Organization report indicates, represents a critical public health challenge worldwide as it accounts for over 720,000 deaths annually, ranking as the third leading cause of death for people in the age bracket of 15 to 29 years old globally [1]. Across the lifespan, Lovero et al., 2023 [2], report that suicide ranks as the 17th cause of death, but of more concern is that these rates may not be indicative of the actual picture because some suicide cases may go un-reported for reasons like stigma, misclassification, or even a lack of sufficient surveillance systems [3]. Beyond mortality, suicidal ideation and attempts contribute substantially to psychological morbidity, healthcare utilization, and also societal burden [3,4,5]. Despite decades of research, suicide prevention still stands out as a complex, multifactorial, and difficult issue to operationalize in both clinical and community settings [6,7,8]. Risk factors for suicide are diverse and include psychiatric disorders, notably major depressive disorder, bipolar disorder, schizophrenia, and substance use disorders and post-traumatic stress disorder, as well as psychosocial stressors, chronic medical illness, and prior suicide attempts [9,10,11,12,13]. The interaction of these biological, psychological, and social determinants in an essential way emphasizes the urgent need for multifaceted prevention strategies that address both proximal and distal risk factors.

Clinical approaches to suicide prevention have historically and in a broad way encompassed risk assessment, acute intervention, and long-term management [14,15]. In addition, validated suicide risk assessment tools, notably the Columbia-Suicide Severity Rating Scale (C-SSRS), Beck Scale for Suicide Ideation (BSS), and Patient Health Questionnaire-9 (PHQ-9), are widely used in research and practice [16,17,18]. However, their predictive validity and ability to guide intervention decisions remain subjects of ongoing debate, primarily because of conceptual, methodological, and practical limitations that constrain their ability in the predicting of suicide and directing clinical decision-making with sufficient precision [19,20,21]. Consequently, clinical trials have increasingly focused on testing both pharmacological and non-pharmacological interventions aimed at reducing suicidal ideation, behaviors, or risk factors [22,23,24,25,26]. Pharmacological strategies have included antidepressants, mood stabilizers, antipsychotics, and, more recently, rapid-acting agents such as ketamine and esketamine [22,27,28]. On the flip side, non-pharmacological approaches encompass psychotherapies, notably cognitive behavioral therapy, and dialectical behavior therapy [20,29]. While many of these strategies show promise, evidence remains heterogeneous, and safety concerns necessitate careful evaluation [30]. ClinicalTrials.gov, notably the largest publicly accessible registry of clinical studies, provides a unique opportunity for the examination of the scope and characteristics of suicide prevention research beyond the published literature alone. The current study was motivated by our observation that beyond the current literature, registry-based analyses have the potential to capture completed studies regardless of publication status in relation to this important topic and, as a result, offer a more comprehensive view of research activity, methodological trends, and reporting practices related to suicide and available interventional approaches. Preliminary research showed that while individual clinical trials provide valuable insights into specific therapeutic approaches, systematic syntheses focusing specifically on completed trials registered on ClinicalTrials.gov remain limited. Capturing completed studies, particularly those with published results, helps mitigate publication bias, offering a comprehensive view of global research activity, structural design trends, and trial reporting practices. Further, this study was further justified by the researchers’ observation through preliminary research that despite the extensive number of suicide-related trials registered over time, few studies have systematically synthesized registry data to evaluate trial design, intervention focus, outcome measures, and reporting completeness. In this context, the core objective here was to analyze completed interventional clinical trials registered on ClinicalTrials.gov which were solely focused on investigating suicide risk assessment, prevention strategies, or related interventions and, through the synthesizing of trial characteristics, populations at risk, validated outcome measures, intervention effectiveness, and safety reporting, identify strengths, limitations, and evidence gaps to inform future suicide prevention research and policy.

## 2. Methodology

### 2.1. Study Design

This registry-based systematic analysis followed the principles of the PRISMA reporting framework for study identification and selection. All data were obtained from publicly available registry records, and no individual participant-level data were accessed. No sample size calculation was performed because this study was a registry-based analysis of completed clinical trials. All eligible studies that met the predefined inclusion criteria were included in the analysis.

### 2.2. Data Source and Search Strategy

ClinicalTrials.gov was searched on 28 March 2026 using the keywords “suicide”, “suicidal ideation”, “suicide prevention”, and “suicide risk assessment”. Searches were limited to completed interventional studies involving adult participants. No restrictions were applied regarding country or year of registration. The limiting of the data source to ClinicalTrials.gov only was necessitated by the observation that this database contains more trials on various topics than any other database, allowing for access to sufficient trials to make meaningful conclusions on the study topic.

### 2.3. Eligibility Criteria

Studies were included if they met all of the following criteria: (1) were registered on ClinicalTrials.gov; (2) had an interventional study design; (3) had a recruitment status listed as completed; and (4) consisted of an evaluation of suicide risk assessment, suicidal ideation, suicidal behavior, or suicide prevention interventions. For clarity, registration on ClinicalTrials.gov was the first check because this study focused solely on this database as the data source. For the trials meeting this registration check, the other conditions had to be met, including having completed and published results.

**Figure 1:**
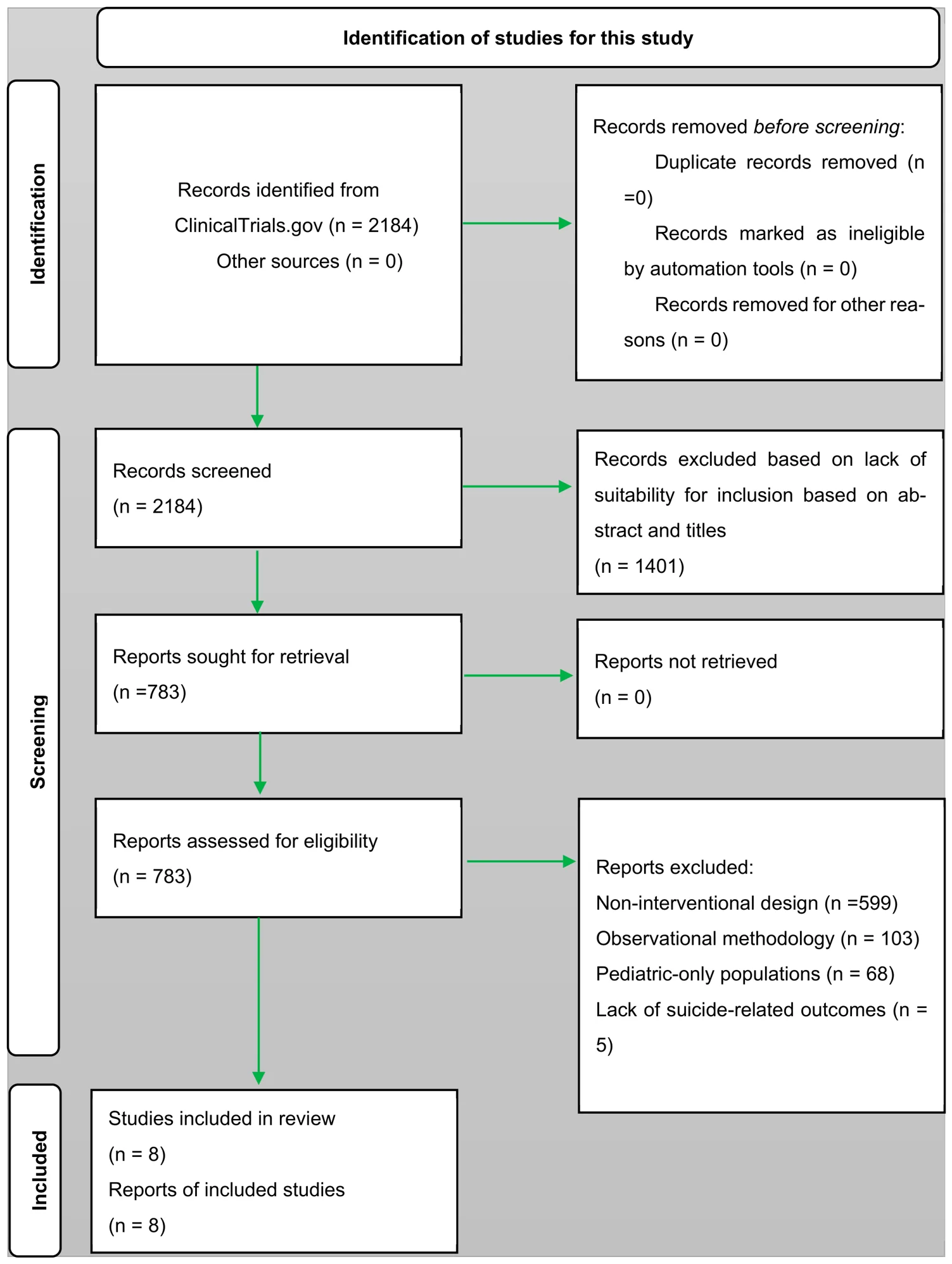
PRISMA chart for selection of trials.

Studies were excluded if they were non-interventional studies or pediatric-only studies or did not include suicide-related outcomes (see Figure 1).

### 2.4. Study Selection Process

Retrieved records (initially a total of 2184) were screened based on titles and registry summaries, followed by full registry review for eligibility confirmation. Reasons for exclusion were documented. The selection process is summarized in a PRISMA-style flow diagram.

### 2.5. Data Extraction, Data Synthesis, and Analysis

Randomization methods were extracted from each ClinicalTrials.gov record when reported, including allocation type, intervention model, and assignment method. Extracted variables included trial phase, design, sample size, population characteristics, suicide risk assessment tools, intervention type, comparator, primary and secondary outcomes, study duration, and safety reporting. We then synthesized the data descriptively, primarily by using narrative summaries and tabulation. Due to heterogeneity in study designs and outcome measures, we did not engage in a meta-analysis of the data. Masking (blinding) status was extracted from each ClinicalTrials.gov record when available (e.g., open-label, single-blind, double-blind, or not reported). No blinding procedures were performed by the authors because this study was based exclusively on publicly available registry data.

### 2.6. Statistical Analysis

Data extracted from the ClinicalTrials.gov registry were summarized using descriptive statistics. Categorical variables are presented as frequencies and percentages, whereas continuous variables are summarized descriptively. Because of the heterogeneity in study designs, interventions, outcome measures, and participant populations, no meta-analysis or inferential statistical analyses were performed. Data extraction, organization, and descriptive analyses were performed using Microsoft Excel (Microsoft Corporation, Redmond, WA, USA).

### 2.7. Ethical Considerations

With this being a study in which the data we collected and analyzed was publicly available and in which there was no access to identifiable patient information, ethical approval was not required. However, we observed all ethical standards that guide research work to ensure the accuracy, validity, and reliability of the findings and conclusions of this study.

### 2.8. Preregistration Statement

This study was not prospectively preregistered as a systematic review protocol. However, all analyzed studies were identified from the publicly accessible clinical trial registry ClinicalTrials.gov, and trial identifiers (NCT numbers) are reported in this manuscript.

## 3. Results

### 3.1. Study Selection

In the initial search of ClinicalTrials.gov (http://clinicaltrials.gov/), we successfully identified a total of 2184 records directly related to suicide, suicidal ideation, suicide risk assessment, and prevention interventions. Upon screening titles and registry abstracts, we excluded a total of 1401 records as we unanimously were convinced that these records were not suitable in providing the information we sought to make conclusions in this study. Full registry entries for 783 studies were reviewed in detail, and after further evaluation, several studies were excluded for non-interventional design, observational methodology, pediatric-only populations, and a lack of suicide-related outcomes, ultimately resulting in 8 completed interventional clinical trials that met all predefined inclusion criteria. The study identification and selection process is summarized in a PRISMA-style flow diagram (Figure 1).

### 3.2. Characteristics of Included Trials

The included trials were predominantly randomized controlled studies (n = 5, approx. 63%), and most were interventional studies (63%). Study designs included individual-level randomization, cluster randomization, and pragmatic care-delivery interventions embedded within the healthcare system. Over one half (n = 5, approx. 63%) of the included trials had their sample sizes reported as fewer than 300 individuals. The largest reported sample size was 18,882 individuals in one trial. Most trials focused on short- to medium-term outcomes. The main characteristics of the eight included trials are summarized in Table 1.

### 3.3. Populations at Risk

All included trials enrolled adult populations who identified as being at elevated risk for suicide. The most commonly studied populations included individuals with major depressive disorder, bipolar disorder, recent psychiatric hospitalization, or prior suicide attempts. Several trials targeted individuals presenting with acute suicidal ideation in emergency, inpatient, or outpatient psychiatric settings, while others focused on high-risk individuals identified through systematic screening in trauma care or primary care environments. Demographic and clinical characteristics substantially varied across studies, notably with the detailed reporting of comorbid psychiatric conditions, psychosocial stressors, and prior treatment history emerging as inconsistent across registry entries.

### 3.4. Interventions and Comparators

The included trials evaluated a diverse range of suicide prevention strategies, encompassing both pharmacological and non-pharmacological approaches. Non-pharmacological interventions predominated and included brief cognitive behavioral therapy, collaborative care models, structured safety planning, brief intervention and contact programs, digital or telehealth-based interventions, and video-based psychoeducational tools. These interventions were typically delivered as adjuncts to standard psychiatric care. Pharmacological interventions were less frequently represented and were primarily evaluated within broader psychiatric treatment frameworks rather than as standalone suicide-specific therapies. Comparators across trials were most commonly treatment as usual, standard care, and present-centered therapy.

### 3.5. Suicide Risk Assessment and Efficacy Outcomes

Across the included trials, efficacy assessment focused on changes in suicidal ideation, suicide attempts, or validated suicide risk scores. The most frequently used assessment instruments included the C-SSRS, BSS, PHQ-9, and clinician-reported suicide attempts or emergency presentations. Outcome designation varied across studies, with some trials specifying suicide-related outcomes as primary endpoints and others including them as secondary or exploratory measures. The timing of assessments also differed, with most trials conducting baseline evaluations followed by repeated assessments at regular intervals during treatment and follow-up. Registry summaries generally indicated whether interventions resulted in reductions in suicidal ideation or behavior compared with comparators; however, detailed numerical results and effect sizes were inconsistently reported, making it difficult to include these details in the current study.

### 3.6. Safety and Tolerability

Safety reporting was present in the majority of included trials, but what is worth noting here is that this varied in depth and completeness. For non-pharmacological interventions, safety monitoring primarily focused on worsening suicidal ideation, adverse psychological responses, or the need for escalation of care. Serious adverse events were infrequently reported, although the absence of standardized safety reporting limited cross-trial comparison, and in trials involving pharmacological or intensive clinical interventions, adverse events were reported inconsistently, and long-term safety data were also highly limited. Most interventions were generally well tolerated, though comprehensive safety profiles were often not fully detailed as captured in the details above.

## 4. Discussion

The objective of the current registry analysis was to provide a comprehensive overview of completed interventional clinical trials evaluating suicide risk assessment and prevention strategies. The findings do highlight key methodological trends, therapeutic approaches, outcome measures, and reporting practices that collectively characterize the current state of suicide prevention research. Most notably, the results here are communicative of the meaningful progress that has been made in intervention development and also reveal notable and persistent limitations that continue to constrain clinical translation and policy impact.

### 4.1. Principal Findings and Research Landscape

The analysis demonstrates that contemporary suicide prevention research is predominantly centered on randomized controlled trials involving high-risk psychiatric populations, essentially reflecting the appropriate prioritization of individuals most vulnerable to suicidal behavior [12,14]. Most included trials focused on adults with recent psychiatric hospitalization or prior suicide attempts, groups who, when evaluated in the context of literature findings from elsewhere, are well known to carry a substantially elevated risk of suicide [31]. In a significant way, this alignment between epidemiological risk and trial enrollment serves to strengthen the clinical relevance of the evidence base. However, it is vital to point out here that the concentration of trials within narrowly defined clinical populations as emerged in this analysis limits generalizability in a substantial way [32]. Community-based populations, primary care patients, and individuals experiencing subthreshold or situational suicidality were underrepresented across all reviewed trials. In light of the current literature and research findings indicating that many individuals who die by suicide have had recent contact with non-specialist healthcare services [32], the limited inclusion of broader populations represents a critical gap in current research efforts, importantly indicating a need for attention to be accorded in this direction in future research works related to this topic.

### 4.2. Intervention Strategies and Therapeutic Focus

A notable finding of this analysis is the predominance of non-pharmacological interventions in suicide-related cases, particularly brief cognitive behavioral therapy, collaborative care models, safety planning interventions, and structured contact-based approaches, again in good alignment with research findings elsewhere [25,26]. Evaluated within the spectrum of impact and the usefulness they have in relation to suicide prevention, it is notable that these strategies emphasize skill-building, crisis management, and continuity of care, essentially rather than reliance on medication alone [33]. In addition, digital and telehealth-based interventions were also featured among the included trials, fundamentally pointing to the broader shifts in mental health service delivery as these modalities offer potential advantages in scalability, accessibility, and continuity for individuals in underserved or rural settings [34]. These virtual and remote modalities offer profound structural advantages over traditional in-person care, particularly regarding scalability, clinical accessibility, and therapeutic continuity. Through the dismantling of geographic and socioeconomic barriers, digital frameworks provide a critical lifeline for individuals residing in underserved or rural settings, who have historically faced severe shortages of localized psychiatric resources. Furthermore, the integration of these technologies not only optimizes clinician workflows but also empowers patient autonomy through asynchronous support tools, ultimately signaling a more resilient, decentralized future for public mental health infrastructure [35]. However, while feasibility and acceptability were frequently reported, robust evidence regarding long-term effectiveness and risk reduction remains limited within the current clinical registry.

Pharmacological interventions were less commonly represented and were typically embedded within broader psychiatric treatment frameworks rather than evaluated as suicide-specific therapies. In our evaluation of this outcome, this result is fundamentally reflective of ethical and methodological challenges associated with pharmacological trials targeting suicidality, most notably including aspects like safety concerns and regulatory constraints [28]. Nonetheless, given emerging evidence for rapid-acting agents in reducing suicidal ideation, future registry analyses may observe increased pharmacological innovation in this area [22].

### 4.3. Outcome Measures and Assessment Challenges

Across the reviewed trials, validated suicide risk and ideation instruments, specifically the Columbia-Suicide Severity Rating Scale, BSS, and PHQ-9 item 9, were widely employed. The use of standardized tools, just as is well reflected within the background literature forming the foundation of the current study, represents a methodological strength in a special way and supports comparability across studies at a conceptual level [15,16,18]. Importantly, the consistent inclusion of patient-reported outcomes acknowledges the subjective and experiential dimensions of suicidality. Despite this, substantial heterogeneity in outcome designation, assessment timing, and reporting granularity was observed, where some trials prioritized suicidal ideation as a primary endpoint, while others treated it as a secondary or exploratory outcome. Additionally, the absence of consistent definitions for clinically meaningful change complicates the interpretation of intervention effectiveness, and in our view these variations essentially serve to limit the ability to draw firm conclusions regarding comparative efficacy and underscore the need for consensus on core outcome sets in suicide prevention research.

### 4.4. Safety Reporting and Ethical Considerations

Safety and tolerability are of paramount importance in suicide prevention trials, yet in our analysis here, we observed notably significant inconsistent and often incomplete safety reporting across registry data [20]. While serious adverse events were infrequently reported, registry entries in several instances did lack detailed descriptions of monitoring procedures, adverse event classification, or escalation protocols, and the authors view this limitation as a particularly concerning aspect given the vulnerable nature of trial populations and the potential for interventions to inadvertently exacerbate distress or risk [7]. Non-pharmacological interventions were generally described as well tolerated, but the systematic reporting of adverse psychological effects was uncommon. Notably, the limited availability of long-term safety data, in our view, further restricts the understanding of sustained intervention impact. Taking into consideration that suicide risk often fluctuates over time, extended follow-up periods and transparent safety reporting should be considered essential components of future trials [4].

### 4.5. Methodological and Reporting Limitations

This study also pinpoints the broader methodological challenges inherent in suicide prevention research. Diagnostic heterogeneity, the inconsistent reporting of comorbidities, and limited demographic detail were common across registry entries. Such gaps hinder the nuanced interpretation of treatment effects and obscure the potential moderators of intervention response, including age, gender, socioeconomic status, and cultural context. Incomplete result reporting within ClinicalTrials.gov emerged as a significant concern. Several completed trials lacked posted results or provided only high-level summaries without numerical data. While trial registration has improved transparency, incomplete compliance with result dissemination undermines the utility of registries as comprehensive research resources. Strengthening the enforcement of reporting requirements is essential to ensure that completed trials meaningfully contribute to the evidence base.

The current study was particularly limited by the sole use of ClinicalTrials.gov as the only data source. While as justified earlier this source contains plenty of accurate and reliable data, other relevant trials could also be registered in other databases. Accordingly, future research on the topic should focus on going beyond ClinicalTrials.gov as the only source of data for reviewed trials. Further, most of the included trials did not have sufficient information to permit risk of bias assessment, which is also a notable limitation of this study.

## 5. Conclusions, Clinical and Policy Implications, and Future Directions

Despite the above limitations, the findings of this analysis have vital implications where, from a clinical standpoint, the results in a great way reinforce the importance of integrated, multimodal suicide prevention strategies tailored to individual risk profiles. The emphasis on behavioral and psychosocial interventions aligns with current clinical guidelines advocating for comprehensive risk management rather than reliance on single interventions. Nevertheless, the modest and variable effectiveness reported across trials points to the considerably urgent need for realistic expectations and shared decision-making in clinical practice. For policymakers and healthcare systems, in line with these findings, it is our view that the prominence of collaborative care and screening-based interventions underscores the potential value of system-level approaches to suicide prevention. Embedding risk assessment and intervention pathways within routine care settings may offer scalable solutions, though effectiveness depends on adequate implementation and resource allocation. Importantly, the fact that only eight studies met the final eligibility criteria was a result of the highly selective screening process. From the initial search, a total of 2184 trials emerged, and 783 were subjected to detailed review, but with the central focus on studies where suicide risk or its ideation, suicidal behavior, and the prevention of it constituted the primary intervention target, the small number of included studies is hereby a reflection of the application of this stringent eligibility criterion.

Looking into the future, we recommend that upcoming suicide prevention research work should aim to address several key gaps identified in this analysis, notably including expanding trial populations beyond narrowly defined psychiatric cohorts, standardizing outcome measures and reporting practices, and extending follow-up durations to assess the durability of intervention effects. In addition, greater emphasis on implementation science, real-world effectiveness, and equity considerations, in our view, will be essential for the purpose of supporting the effective translation of research findings into meaningful reductions in suicide risk.

## Figures and Tables

**Table 1:** Summary data of analyzed trials.

Trial Identifier (NCT)	Study Phase/Type	Study Design	Sample Size (n)	Population Characteristics	Intervention	Comparator	Primary Suicide-Relevant Outcomes	Study Duration
NCT04844294	Interventional RCT	Randomized, parallel	98	Adults with suicidal ideation and/or behavior	Brief cognitive behavioral therapy (telehealth)	Present-centered therapy (PCT)	Suicide attempts; severity of suicidal ideation	12-month follow-up
NCT02655354	Pragmatic Trial	Cluster-randomized pragmatic trial	635	Trauma survivors with suicidal ideation (PHQ-9 screening)	Collaborative care intervention	Control/usual care	Suicidal ideation (PHQ-9 item-9); suicide monitoring	3-12-month follow-up
NCT03825354	Pilot RCT	Pragmatic randomized	61	Adults at elevated suicide risk	Brief intervention and contact (BIC) program	Treatment as usual	Feasibility; future suicide attempts	Registry summary
NCT04307394	Interventional	Open-label or randomized	10	Recently hospitalized psychiatric patients	Adjunctive video-based suicide prevention	Usual care	Suicidal ideation; repeat attempts	Registry summary
NCT05530941	Interventional	Behavioral intervention	205	Adults with suicide/self-harm risk	Online suicide prevention campaign	None/standard	Help-seeking behaviors; ideation	Registry summary
NCT06130943	Interventional	Randomized/controlled	Not reported	Adults with suicidal ideation	Suicide ideation prevention strategies	Comparator as per protocol	Suicidal ideation scale changes	Registry summary
NCT02326883	Network Trial	Interventional care management	18,882 (EHR-based)	Elevated suicide risk in healthcare settings	Suicide risk screening + referral	Usual care	CSSRS screening; referral outcomes	Registry/NIH sources
NCT04168645	Interventional	CBT for post-discharge	213	Psychiatric inpatients post-suicide attempt	Inpatient CBT	Treatment as usual	Suicidal behavior post-discharge	Registry

Note: NCT04844294 is the ClinicalTrials.gov identifier of one included trial analyzed in this registry-based review. It is not the clinical trial registration number of the present study. Therefore, the clinical trial number for the present study is not applicable.

## Data Availability

No new data were created or analyzed in this study. Data sharing is not applicable to this article.

## Abbreviation

Abbreviation	Full Term
BIC	Brief Intervention and Contact
BSS	Beck Scale for Suicide Ideation
CBT	Cognitive Behavioral Therapy
C-SSRS	Columbia-Suicide Severity Rating Scale
EHR	Electronic Health Record
NCT	National Clinical Trial Identifier
PHQ-9	Patient Health Questionnaire-9
PCT	Present-Centered Therapy
PTSD	Post-Traumatic Stress Disorder
RCT	Randomized Controlled Trial
WHO	World Health Organization
